# Chlorination Disinfection By-Products and Risk of Congenital Anomalies in England and Wales

**DOI:** 10.1289/ehp.10636

**Published:** 2007-11-06

**Authors:** Mark J. Nieuwenhuijsen, Mireille B. Toledano, James Bennett, Nicky Best, Peter Hambly, Cornelis de Hoogh, Diana Wellesley, Patricia A. Boyd, Lenore Abramsky, Nirupa Dattani, John Fawell, David Briggs, Lars Jarup, Paul Elliott

**Affiliations:** 1 Small Area Health Statistics Unit, Department of Epidemiology and Public Health, Faculty of Medicine, Imperial College London, St. Mary’s Campus, London, United Kingdom; 2 Center for Research in Environmental Epidemiology (CREAL), Barcelona, Spain; 3 British Isles Network of Congenital Anomaly Registers, BINOCAR Management Committee, Newcastle-Upon-Tyne, United Kingdom; 4 National Perinatal Epidemiology Unit, University of Oxford, Oxford, United Kingdom; 5 North Thames Perinatal Public Health Unit, Northwick Park Hospital, Harrow, United Kingdom; 6 Office for National Statistics, London, United Kingdom; 7 Independent Consultant, High Wycombe, Buckinghamshire, United Kingdom

**Keywords:** chlorination, congenital anomalies, disinfection by-products, trihalomethanes

## Abstract

**Background:**

Increased risk of various congenital anomalies has been reported to be associated with trihalomethane (THM) exposure in the water supply.

**Objectives:**

We conducted a registry-based study to determine the relationship between THM concentrations and the risk of congenital anomalies in England and Wales.

**Methods:**

We obtained congenital anomaly data from the National Congenital Anomalies System, regional registries, and the national terminations registry; THM data were obtained from water companies. Total THM (< 30, 30 to < 60, ≥60 μg/L), total brominated exposure (< 10, 10 to < 20, ≥20 μg/L), and bromoform exposure (< 2, 2 to < 4, ≥4 μg/L) were modeled at the place of residence for the first trimester of pregnancy. We included 2,605,226 live births, stillbirths, and terminations with 22,828 cases of congenital anomalies. Analyses using fixed- and random-effects models were performed for broadly defined groups of anomalies (cleft palate/lip, abdominal wall, major cardiac, neural tube, urinary and respiratory defects), a more restricted set of anomalies with better ascertainment, and for isolated and multiple anomalies. Data were adjusted for sex, maternal age, and socioeconomic status.

**Results:**

We found no statistically significant trends across exposure categories for either the broadly defined or more restricted sets of anomalies. For the restricted set of anomalies with isolated defects, there were significant (*p* < 0.05) excess risks in the high-exposure categories of total THMs for ventricular septal defects [odds ratio (OR) = 1.43; 95% confidence interval (CI), 1.00–2.04] and of bromoform for major cardiovascular defects and gastroschisis (OR = 1.18; 95% CI, 1.00–1.39; and OR = 1.38; 95% CI, 1.00–1.92, respectively).

**Conclusion:**

In this large national study we found little evidence for a relationship between THM concentrations in drinking water and risk of congenital anomalies.

Since chlorination disinfection by-products (DBPs) were first reported in drinking water (Rook 1974), there have been concerns about potential adverse reproductive health effects, including low birth weight, spontaneous abortion, stillbirth, and congenital anomalies (Nieuwenhuijsen et al. 2000a), but findings of the studies to date have been inconsistent. Statistically significant positive associations have been reported between trihalomethane (THM) exposure and neural tube defects (NTDs) (Bove et al. 1995; Dodds and King 2001; Klotz and Pyrch 1999), major cardiac defects (Cedergren et al. 2002; Hwang et al. 2002), urinary tract defects (Aschengrau et al. 1993; Hwang et al. 2002; Magnus et al. 1999), and respiratory defects (Aschengrau et al. 1993; Hwang et al. 2002), whereas other studies did not find such associations (Dodds et al. 1999; Källén and Robert 2000; Shaw et al. 2003). Studies on oral cleft or cleft palate have largely been negative, except for the study by Bove et al. (1995). Only Dodds and King (2001) and Shaw et al. (2003) studied the effect of specific THMs. Dodds and King (2001) found a statistically significant association between bromodichloromethane (BDCM) and NTDs, whereas Shaw et al. (2003) found a few statistically significant negative associations with NTDs and cleft lip and palate. One of the main limitations in most of these studies has been small sample size, resulting in low power to explore exposure–response relationships.

In the present study, the largest study of its kind so far, we report the relationships between THM levels in the public water supply and risk of congenital anomalies across England and Wales. Primary analyses focused on total THM and broad categories of congenital anomalies; secondary analyses focused on restricted subsets of anomalies and specific THM groups, including bromoform and brominated THMs.

## Methods

### Study region and years

**Figure 1:**
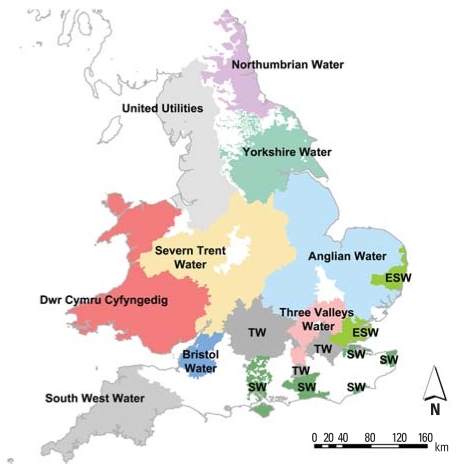
Regions covered by water companies included in the analyses. Abbreviations: ESW, Essex and Suffolk Water; SW, Southern Water; TW, Thames Water. The white areas were not included in the study.

The study area was covered by 12 water companies in the United Kingdom: United Utilities (formerly North West; estimated population, 6.8 million), Severn Trent (7.6 million), Northumbrian (2.6 million), Anglian Water (4.0 million), Bristol (1 million), Dwr Cymru Cyfyngedig (3 million), Essex and Suffolk (1.7 million), Southern (2.2 million), South West (1.3 million), Thames Water (7.4 million), Three Valleys (2.4 million), and Yorkshire (4.2 million) (Figure 1). Under regulations in force at the time the THM samples were taken, water companies divided their water supply into water supply zones, each zone covering a population of < 50,000 people. Less than 1% of households in the United Kingdom have private water supplies. We chose this study area because it had considerable variation in THM concentrations between water zones, and digital water-zone boundaries were available within geographic information systems (GIS). GIS data were available for Northumbrian for 1997, United Utilities for 1993–1997, and Severn Trent for 1993–1998 from a previous study in these areas (Toledano et al. 2005), and for all of the companies for 1998–2001; these data were directly provided by the water companies and checked by the study team. The main method of disinfection during the study period was chlorination, with a few regions having additional chloramination. Ozone was often used primarily for removal of organic material, but it also would have acted as a disinfectant.

### Exposure data

We used THM concentrations as the marker for chlorine disinfection by-products. Water samples were routinely collected and analyzed from each water zone using random samples from each consumer’s tap. Under regulations in force at the time, the standard sampling frequency for THMs was a minimum of four samples per year. However, if there was a breach of the standard of 100 μg/L for total THMs, the sampling frequency increased to a minimum of 12 or 24 per year, depending on the zone size. Conversely, if the total THM concentration was consistently < 50% of the standard, a reduced frequency of at least one per year could have been used.

Because of the small number of THM measurements in some water zones, the need for quarterly (3 month) estimates (to allow for trimester-weighted exposure estimates), and the problem of measurements below the limit of detection (LOD), it was necessary to model the raw THM data to obtain more robust estimates of the mean THM concentration in each zone. This was done using a hierarchical mixture model in the software WinBUGS (Bayesian inference using Gibbs sampling) (Spiegelhalter et al. 1996), as described elsewhere (Whitaker et al. 2005). Briefly, modeling was carried out separately for each water company and year. The model calculated the mean annual individual THM concentrations for each water zone and subsequently assigned an estimated water source type to each water zone, depending on the four THM levels within each zone. We fitted a three-component mixture model in which zones were assumed to belong to one or some mixture of three components that we labeled “ground,” “lowland surface,” and “upland surface” waters (the components may not strictly correspond to these three water source types; we simply aimed to group waters with similar THM profiles, which are more likely to be shared among water of the same source type). The hierarchical model enabled zones to “borrow” information from other zones with the same water source type. This resulted in more stable estimates for zones where few samples were taken. For measurements below the LOD, we used our model to obtain an estimate between zero and the LOD (rather than arbitrarily assigning one-half or two-thirds the LOD, which is common practice). We took into account seasonal variation by estimating a quarterly effect common to all zones supplied by the same source type.

### Congenital anomaly data

**Figure 2:**
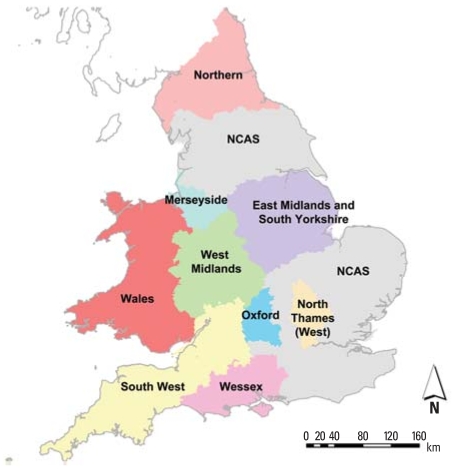
Regions covered by regional congenital anomalies registries.

Individual post-coded records were extracted from the national births, stillbirths, terminations, and congenital anomalies registers [National Congenital Anomalies System (NCAS)] held at the UK Small Area Health Statistics Unit (SAHSU). In addition, individual postcoded records were obtained from the regional registries via the British Isles Network of Congenital Anomaly Registers (BINOCAR), which covers about 50% of the population of England and Wales (Figure 2). We merged the data from the national and regional registries to obtain one numerator database. Duplicate records across data sets were removed, with regional registry records prioritized. We used national and regional registry region boundaries, as defined by the Office for National Statistics, to delineate NCAS and BINOCAR regions. The main analyses focused on broad categories of congenital anomalies: cleft lip/palate {*International Classification of Diseases, 10th Revision* [ICD-10; World Health Organization (WHO) 1992] codes Q35–Q37}; diaphragmatic hernia and abdominal wall defects (Q79); major cardiac defects (Q20–Q28); NTDs (Q00, Q01, and Q05); urinary tract defects (Q60–Q64); and respiratory defects (Q30–Q34).

We conducted further analyses using restricted groups of congenital anomalies that were considered to be etiologically coherent, with better ascertainment, defined as follows: abdominal wall defects (ICD-10 codes Q79.0, Q79.1, Q79.2, and Q79.3), major cardiac defects (Q20, Q21.2, Q21.3, Q22, Q23, Q25.1–Q25.9, and Q26), urinary tract defects (Q60, Q61, Q62, and Q64, but excluding Q62.0, Q64.8, and Q64.9), and respiratory defects (Q33). In addition we conducted separate analyses for cleft palate (ICD-10 code Q35), cleft lip with and without cleft palate (Q36–Q37.99), exomphalos (Q79.2), gastroschisis (Q79.3), hypoplastic left heart syndrome (Q23.4), ventricular septal defects (Q21.0), and two subsets of urinary tract defects including renal disease (Q60 and Q61), and obstructive disease (Q62 and Q64). We also included congenital anomalies of the esophagus (Q39) in these analyses. For earlier years (before 1995), we bridged ICD-9 (WHO 1977) and ICD-10 (WHO 1992) codes manually. We conducted further analyses excluding cases with anomalies that were found to be part of a chromosomal syndrome (i.e., chromosomal anomalies, any mention of ICD-10 code Q9), as well as examining those cases with isolated anomalies only.

The number of anomalies recorded per baby was known to vary across BINOCAR regions, with some regional registries more likely to register a more detailed number of minor anomalies for each baby than others. We did not consider it appropriate, therefore, to classify all babies registered with one anomaly as “isolated” and all those registered with more than one anomaly as having “multiple anomalies.” Instead, we devised an isolated/multiple classification that we judged to be largely unaffected by differential reporting of minor anomalies. First, we compiled a list of all other major anomalies not already included in the study [i.e., ICD-10 codes Q11*, Q12*, Q13.0, Q41*, Q42*, Q54*, Q55.5, Q55.6, Q66.0, Q68*, Q71*, Q72*, Q89.3 (“*” indicates any subcategory of the code)]. Classification was then undertaken for all broad and restricted category analyses as follows (with the exception of abdominal wall defects): babies were classified as “multiple” if they had more than one anomaly that fell into more than one of the six broad categories (i.e., cleft lip/palate, abdominal wall, major cardiac, NTD, urinary, respiratory) or if they had one (or more) anomalies from one of the six broad categories together with one (or more) from the list of additional major anomalies above. For abdominal wall anomalies and gastroschisis (ICD-10 code Q79.3) and exomphalos only (Q79.2), classification of “multiple” was as described above, with the addition that, if more than one anomaly was registered from within the same broad abdominal wall category (ICD-10 code Q7*), the baby was also classified as having “multiple” anomalies. Thus, for a baby to be classified as having an “isolated” abdominal wall anomaly, there could be only one abdominal wall code.

There was a total of 22,828 cases with congenital anomalies; 1,641 (7.2%) of these had a chromosomal defect, 2,249 (9.9%) were classified as having multiple (nonchromosomal) anomalies, and 18,938 (83.0%) were classified as having isolated anomalies only.

The study population was defined according to the first possible date on which THM data for the first trimester was available (i.e., 15 October 1993 for United Utilities and Severn Trent; 15 October 1997 for Northumbrian; and 15 October 1998 for all other water regions) until 31 December 2001.

### GIS methods and data linkage

We created a postcode to water-zone link using point-in-polygon methods within the GIS to allocate each postcode to its water supply zone. Postcode locations were derived from the historical postcode file for Great Britain, developed by SAHSU. This file traces post-codes back in time and assigns a grid coordinate for each postcode in each year. To take account of changes in the location of both postcodes and water-zone boundaries over time, we created a separate link for each year of the study period.

We used the postcode of the maternal residence at the year of birth to identify the water zone of interest and hence the appropriate exposure status for each birth record. The latter was obtained by first calculating a weighted average of the modeled quarterly THM estimates for the appropriate zone for the first 93 days of the pregnancy. For cases, a gestational age was generally available and the first 93 days of pregnancy was calculated. Where gestational age was missing, we assigned the anomaly-specific average gestation weeks. The weighting was based on the proportion of the trimester falling into each quarterly period. Where the pregnancy was < 93 days, for example, for terminations, we used the whole pregnancy time period. For noncases, we had to assume that births had gone to term when calculating the first 93 days of the pregnancy, as data on gestation weeks at birth are not recorded on the birth records. Finally, the weighted average THM estimate associated with each birth record was categorized into one of three predefined exposure categories: concentrations of total THMS (TTHMs; < 30, 30 to < 60, and ≥60 μg/L), total brominated THMs (< 10, 10 to < 20, and ≥20 μg/L), and bromoform (< 2, 2 to < 4, and ≥4 μg/L). These were chosen with reference to the published literature on the possible associations of birth outcomes with THMs and with regard to the joint distribution of numbers of births and THM concentrations across the water regions.

### Exclusions and study size

We excluded a total of 49,558 pregnancy outcomes, including 377 with congenital anomalies. The main reasons for exclusion were either that no exposure estimates were available and/or that lagging for the critical exposure period was not possible. This left 2,605,226 births for analysis, including live births, stillbirths, and terminations.

### Statistical analyses

Using the statistical package R, we carried out descriptive analysis and univariate and multiple logistic regression modeling with adjustment for the following potential confounders: *a*) maternal age (for which individual level information was available) categorized as < 21, 21–25, 26–30, 31–35, and > 35 years; *b*) socioeconomic status categorized into quintiles of an areal deprivation index (Carstairs and Morris 1991) according to location of the postcode of maternal residence at the time of birth, using a combination of four indicators at the level of 2001 census output area (percentage of people with no car, in overcrowded housing, with head of household in social class IV or V, and percentage of men unemployed); *c*) year of birth; and *d*) registry (BINOCAR or NCAS region). Interactions between THM exposure and potential confounding variables were tested where appropriate.

We conducted analyses by individual BINOCAR/NCAS region, and tested for heterogeneity of risks associated with THM exposure across the BINOCAR/NCAS regions. We conducted analyses using both fixed-effects and random-effects models. Where there was significant (*p* < 0.05) heterogeneity, we used results of the random-effects model to obtain an overall summary estimate of the effect of THM, allowing for heterogeneity in the region-specific estimates (Dersimonian and Laird 1986). Where there was no evidence of heterogeneity, we present the fixed-effects models.

## Results

### Descriptive statistics

Tables 1 and 2 describe THM concentrations by exposure categories and the correlation of the various individual THMs. Mean TTHM concentrations ranged from 16.4 μg/L in the low-exposure category to 72.2 μg/L in the high-exposure category. We observed the highest correlations between total brominated THMs and dibromochloromethane (0.93), and between TTHM and chloroform (0.90).

The prevalence of each broad congenital anomaly group by deprivation, sex, maternal age, and BINOCAR/NCAS region is shown in Table 3. The number of anomalies ranged from 1,434 for respiratory defects to 8,809 for major cardiac defects. We found higher prevalence of each anomaly when we compared the most deprived areas to the most affluent areas. Prevalence in males and females was similar, except for cleft lip/palate and urinary tract defects, where prevalence was 50–100% higher in males. We observed U-shaped relationships between prevalence of congenital anomalies and maternal age, except for NTDs where the prevalence decreased with increasing maternal age. The reported prevalence of each anomaly was substantially higher in the BINOCAR regions than in the NCAS regions, reflecting better ascertainment. Rates also varied among the regional registries (data not shown).

### Regression models

**Figure 3:**
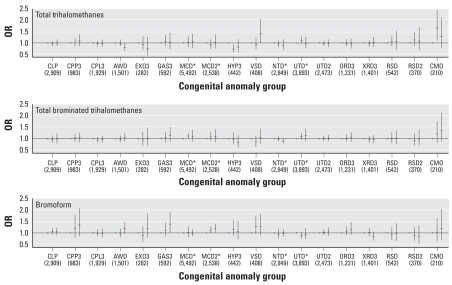
Adjusted overall ORs and 95% CIs (number of cases in each anomaly group) for various isolated congenital anomalies by TTHM categories (low, < 30; medium, 30 to < 60; high, ≥60 μg/L), total brominated THM categories (low, < 10; medium, 10 to < 20; high, ≥20 μg/L), and bromoform categories (low, < 2; medium, 2 to < 4; high, ≥4 μg/L) in England and Wales, 1993–2001. Abbreviations: CLP, broad category of cleft lip and palate; CPP3, restricted group of cleft lip and palate with cleft lip; CLP3, restricted group of cleft palate; AWD, abdominal wall defects; EXO3, exomphalos; GAS3, gastroschisis; MCD, major cardiac defects; MCD2, restricted group of major cardiac defects; HYP3, hypoplastic left heart syndrome; VSD, ventricular septal defects; UTD, broad category of urinary tract defects; UTD2, restricted category of urinary tract defects; ORD3, obstructive urinary defects; XRD3, all renal defects; RSD, broad category of respiratory defects; RSD2, restricted category of respiratory defects; CMO, congenital anomaly of the esophagus. ORs are presented for the medium- and high-exposure categories, with the low-exposure category as the reference group. Overall summary estimates were obtained by combining the registry-specific exposure ORs using a fixed-effects model (where there was no evidence of heterogeneity), adjusted for deprivation quintile, year, water company, and maternal age. *Denotes use of a random-effects model that allowed for heterogeneity between registries where indicated.

Unadjusted (data not shown) and adjusted analyses showed similar risk estimates. We found no statistically significant trends across the three exposure categories for total THMs, total brominated THMs, or bromoform for either the broadly defined or more restricted sets of anomalies. The only significant associations (*p* < 0.05) with the broadly defined groups of anomalies was a deficit risk of abdominal wall defects in the high TTHM exposure category [odds ratio (OR) = 0.81; 95% confidence interval (CI), 0.68–0.95] and an excess risk of major cardiac defects in the medium (but not high) exposure category of total brominated THMs (OR = 1.12; 95% CI, 1.01–1.23) (Table 4). For the restricted set of isolated anomalies, we observed statistically significant excess risks for TTHM in the high-exposure category of ventricular septal defects (OR = 1.43; 95% CI, 1.00–2.04) and in the medium- (but not high) exposure category for congenital anomalies of the esophagus (OR = 1.66; 95% CI 1.12–2.45). For bromoform, there was a significant excess in the high-exposure category for both major cardiac defects (OR = 1.18; 95% CI, 1.00–1.39) and gastroschisis (OR = 1.38; 95% CI, 1.00–1.92) (Figure 3).

We found no significant interactions between TTHM exposure and any of the potential confounders. Analyses of cases with multiple anomalies showed no significant association with THM concentrations, but the numbers were small (data not shown). Sensitivity analyses that excluded the NCAS data made little difference to the overall results.

## Discussion

The present study is the largest study to date to examine the relationship between THM exposure and congenital anomalies, and the first to examine the effects of bromoform on congenital anomalies and the effects of THM exposure on gastroschisis. We found little evidence of a relationship between concentrations of THMs and a wide spectrum of congenital anomalies. There were no statistically significant exposure–response trends across the exposure categories for any of the anomalies studied. Statistically significant excess risks were observed for isolated anomalies only for ventricular septal defects and esophageal anomalies in the high- and medium-exposure categories, respectively, of TTHMs and for a subset of major cardiac defects and gastroschisis in the high-exposure category of bromoform. In the context of this study, these may have been chance associations; there is still little or no toxicologic evidence for reproductive or teratogenic effects of bromoform, or other DBPs (Nieuwenhuijsen et al. 2000a); also, the concentrations of bromoform across our study regions were generally very low, with only 19% of the population being exposed to levels > 4 μg/L (high-exposure group). In the only other epidemiologic study reporting bromoform levels, the mean level reported by Savitz et al. (2006) at their brominated DBP site (6.4 μg/L) was similar to the mean in our high-exposure group; mean levels at their other two sites were also low (0.1 and 0.6 μg/L).

In contrast, the careful selection of subsets of major cardiac defects, ventricular septal defects, and gastroschisis as isolated anomalies may have increased accuracy of case definition (and reduced misclassification). Furthermore, Geter et al. (2005) suggested potential epigenetic effects of bromoform and potential mechanisms, such as alteration in DNA methylation, that could result in effects on cell proliferation/apoptosis; they also suggested that increased homocysteine levels could lead to oxidative stress, which also may feed into cell birth/cell death balance. Further study of these specific anomalies and bromoform exposure may be warranted.

Using color as a surrogate for DBP exposure, Hwang et al. (2002) also found significant excess risks of ventricular septal defects. Adjusted ORs were 1.63 (95% CI, 1.02–2.58) and 1.81 (95% CI, 1.05–3.09) for the medium- and high-exposure categories, respectively. Hwang et al. (2002) and Cedergren et al. (2002) found statistically significant associations between major cardiac defects and chlorinated water/concentrations of TTHM > 10 μg/L. Other studies have reported no associations with cardiac defects (Bove et al. 1995; Dodds and King 2001; Dodds et al. 1999; Magnus et al. 1997; Shaw et al. 2003). Unlike the present study, three other studies reported significant positive associations between chlorinated water and urinary tract defects (Aschengrau et al. 1993; Hwang et al. 2002; Magnus et al. 1999), and two of three studies to date have found a significant positive association with respiratory defects (Aschengrau et al. 1993; Hwang et al. 2002). Klotz and Pyrch (1999) found a statistically significant association between TTHM and NTDs, but not with concentrations of haloacetonitriles and haloacetates. Also, the effects were most pronounced in offspring of women who did not take supplementary vitamins, but these findings were not replicated in the study by Shaw et al. (2003). Moreover, inclusion of information on ingestion, showering, bathing, and swimming made little difference to the risk estimates (Klotz and Pyrch 1999). In a meta-analysis, Hwang and Jaakkola (2003) reported a significant association between chlorination by-product exposure and risk of NTDs and urinary system defects, but results for respiratory system, major cardiac, and oral cleft defects were heterogeneous and inconclusive.

Various factors may have contributed to the lack of consistency between studies, including differences in exposure and outcome definitions, case ascertainment, exposure misclassification (due in part to the relatively crude methods of exposure assessment), differences in the composition of DBPs in the water supply, and low statistical power due to small sample size. In the present study we addressed a number of these weaknesses—specifically by paying careful attention to case definition, the use of subsets of anomalies, the large sample size, and the use of modeled trimester-weighted THM exposure estimates—to improve exposure classification. The next largest study included approximately 285,000 births and 5,764 cases of congenital anomalies (Hwang et al. 2002).

One of the main limitations of registry-based studies of congenital anomalies is that ascertainment is geographically variable and often incomplete. In the United Kingdom, the NCAS ascertains only around 40% of the congenital anomalies compared with the BINOCAR registries (Boyd et al. 2005). Similarly, we found a substantial difference in prevalence rates between the NCAS and BINOCAR registries, as well as differences in prevalence rates between the various regional registries, which might in part reflect differences in methods of ascertainment and completeness of reporting (Rankin et al. 2005). In the present study, analysis of the BINOCAR data alone (where case ascertainment was higher) made little difference to the overall results. Variation in reporting rates is unavoidable when registration is not a statutory requirement, unlike, for example, the registration of births or deaths, although some anomalies such as gastroschisis have good and consistent ascertainment across registries. Such geographic variations should not bias study results, provided that completeness of reporting is unrelated to the exposure of interest. We used an isolated/multiple classification among a restricted set of anomalies to overcome differential reporting of minor anomalies, but there was some indication that such differential reporting may have occurred. We found opposing trends between NCAS regions (where ascertainment was highest in the high-exposure categories) and BINOCAR regions (where ascertainment was lowest in the high-exposure categories), although no such trends were apparent for the brominated compounds. Whether such trends reflect differences in case definition or completeness of reporting across registries and exposure categories, and whether these may have led to important biases, is difficult to establish with any certainty.

Further limitations are the lack of information on gestation age and on mobility of women during pregnancy, both of which may have led to exposure misclassification, and hence attenuation in risk estimates.

To take into account heterogeneity between regions served by different registries, we conducted the analyses separately for each registry and used meta-analysis to obtain summary ORs. If THMs are an imperfect proxy for other by-products, then heterogeneity in risk estimates between regions might be expected, and a random-effects model may be most appropriate. If the THMs are the substances of interest, a fixed-effects model may be the most appropriate. In the present study, the choice of model made little difference to magnitude of the risk estimates, although the confidence intervals were slightly wider for random-effects models.

Although one of the main strengths of our study is its size, this and its retrospective nature simultaneously limit the options available for exposure assessment. Whereas the approach used here appears to provide valid estimates of THM exposure for epidemiologic study (Keegan et al. 2001; Nieuwenhuijsen et al. 2000b; Toledano et al. 2005; Whitaker et al. 2003), the lack of association between THMs and congenital anomalies does not preclude the possibility of an association at the individual level, or between other DBPs and congenital anomalies. THM concentrations may not be a good marker of other byproducts (e.g., haloacetates) that have recently been implicated with respect to adverse birth outcomes (Hinckley et al. 2006; Porter et al. 2005; Wright et al. 2004). For example, we reported only a moderate correlation between THMs and haloacetic acids in parts of the study area (Malliarou et al. 2005). However, among > 500 different DBPs that have been identified (Richardson 1998), THMs and haloacetic acids are present in by far the greatest concentrations; others are present at much smaller concentrations, usually < 1 μg/L.

Currently there is no plausible biological mechanism by which chlorination by-products could cause congenital anomalies, particularly at low concentrations. Nonetheless, the policy of minimizing the concentrations of chlorination by-products in the public water supply by removing the natural organic precursors, while simultaneously maintaining the level of protection from disinfection, seems appropriate in view of concerns about possible adverse reproductive health effects (Nieuwenhuijsen et al. 2000a, 2000b). The WHO has continued to emphasize that high levels of protection from disinfection should never be compromised in trying to reduce disinfection by-product concentrations; our data do not detract from that view.

## Correction

In the manuscript originally published online, column titles in Table 4 were incomplete. They have been corrected here.

## Figures and Tables

**Table 1:** Characteristics of the THM exposure categories (μg/L).

	Mean ± SD	Minimum	Maximum	No.^a^	No. of water zones
Total THMs					
Low	16.4 ± 8.7	0.5	< 30.0	1,062,158	1,289
Medium	43.6 ± 8.6	30.0	< 60.0	1,109,346	1,220
High	72.2 ± 10.1	60.0	130.9	433,722	698
Total brominated THMs					
Low	6.6 ± 2.4	0.4	< 10.0	816,863	1,175
Medium	14.2 ± 2.9	10.0	< 20.0	1,187,932	1,345
High	28.3 ± 8.4	20.0	74.9	600,431	828
Bromoform					
Low	0.9 ± 0.5	0.0	< 2.0	1,575,323	1,417
Medium	2.9 ± 0.6	2.0	< 4.0	538,570	1,000
High	6.7 ± 3.2	4.0	51.8	491,333	775

a Number of live births, stillbirths, and terminations.

**Table 2:** Pearson correlations between various species of THMs at water-zone level.

	Bromoform	Chloroform	DBCM	TBROM	TTHM
BDCM	−0.11	0.46	0.50	0.73	0.74
Bromoform		−0.44	0.61	0.54	−0.18
Chloroform			−0.30	−0.05	0.90
DBCM				0.93	0.12
TBROM					0.38

DBCM, dibromochloromethane; TBROM, total brominated species (BDCM, DBCM, and bromoform).

**Table 3:** Prevalence rates (per 1,000) of various congenital anomalies by potential confounding variables.

		Cleft lip/palate		Abdominal wall		Major cardiac		Neural tube		Urinary tract		Respiratory	
	No. of births and terminations	No. of cases	Prevalence (95% CI)	No. of cases	Prevalence (95% CI)	No. of cases	Prevalence (95% CI)	No. of cases	Prevalence (95% CI)	No. of cases	Prevalence (95% CI)	No. of cases	Prevalence (95% CI)
Total	2,605,226	3,736	1.43 (1.39–1.48)	2,267	0.87 (0.84–0.91)	8,809	3.38 (3.31–3.45)	3,334	1.28 (1.24–1.32)	5,315	2.04 (1.99–2.10)	1,434	0.55 (0.52–0.58)
Deprivation quintiles													
1 (affluent)	425,593	602	1.41 (1.31–1.53)	292	0.69 (0.61–0.77)	1,302	1.55 (1.43–1.67)	470	1.10 (1.01–1.21)	777	1.83 (1.70–1.96)	202	0.47 (0.41–0.54)
2	442,019	585	1.32 (1.22–1.44)	300	0.68 (0.61–0.76)	1,419	1.60 (1.48–1.72)	483	1.09 (1.00–1.19)	830	1.88 (1.75–2.01)	203	0.46 (0.40–0.53)
3	475,900	655	1.38 (1.27–1.49)	371	0.78 (0.70–0.86)	1,580	1.74 (1.63–1.86)	603	1.27 (1.17–1.37)	909	1.91 (1.79–2.04)	278	0.58 (0.52–0.66)
4	514,593	729	1.42 (1.32–1.52)	587	1.14 (1.05–1.24)	1,880	1.79 (1.68–1.91)	724	1.41 (1.31–1.51)	1,165	2.26 (2.14–2.40)	328	0.64 (0.57–0.71)
5 (deprived)	747,001	1,165	1.56 (1.47–1.65)	717	0.96 (0.89–1.03)	2,626	1.77 (1.67–1.86)	1054	1.41 (1.33–1.50)	1,633	2.19 (2.08–2.29)	422	0.56 (0.51–0.62)
Registry region													
NCAS	1,060,401	1,020	0.96 (0.90–1.02)	552	0.52 (0.48–0.57)	1,322	1.25 (1.18–1.32)	905	0.85 (0.80–0.91)	1,112	1.05 (0.99–1.11)	174	0.16 (0.14–0.19)
BINOCAR	1,544,825	2,716	1.78 (1.71–1.84)	1,715	1.12 (1.07–1.18)	7,487	4.90 (4.79–5.01)	2,429	1.59 (1.53–1.65)	4,203	2.75 (2.67–2.83)	1,260	0.82 (0.78–0.87)
Maternal age (years)													
< 21	294,135	390	1.33 (1.20–1.46)	516	1.75 (1.61–1.91)	925	3.14 (2.95–3.35)	431	1.47 (1.33–1.61)	635	2.16 (2.00–2.33)	170	0.58 (0.50–0.67)
21–25	550,562	806	1.46 (1.37–1.57)	538	0.98 (0.90–1.06)	1,615	2.93 (2.79–3.08)	746	1.35 (1.26–1.46)	1,122	2.04 (1.92–2.16)	289	0.52 (0.47–0.59)
26–30	819,488	1,034	1.26 (1.19–1.34)	514	0.63 (0.58–0.68)	2,273	2.77 (2.66–2.89)	1,015	1.24 (1.16–1.32)	1,555	1.90 (1.81–1.99)	398	0.49 (0.44–0.54)
31–35	660,909	860	1.30 (1.22–1.39)	410	0.62 (0.56–0.68)	1,903	2.88 (2.75–3.01)	805	1.22 (1.14–1.31)	1,201	1.82 (1.72–1.92)	320	0.48 (0.43–0.54)
> 35	274,106	427	1.56 (1.42–1.71)	253	0.92 (0.82–1.04)	1,109	4.05 (3.82–4.29)	327	1.19 (1.07–1.33)	551	2.01 (1.85–2.18)	197	0.72 (0.63–0.83)
Sex of baby^a^													
Male	1,332,251	1,034	0.78 (0.73–0.82)	408	0.31 (0.28–0.34)	1,232	0.92 (0.87–0.98)	196	0.15 (0.13–0.17)	1,134	0.85 (0.80–0.90)	159	0.12 (0.10–0.14)
Female	1,265,381	722	0.57 (0.53–0.61)	343	0.27 (0.24–0.30)	1,092	0.86 (0.81–0.92)	226	0.18 (0.16–0.20)	539	0.43 (0.39–0.46)	134	0.11 (0.09–0.13)

a Information on sex of baby was unavailable for terminations (2,636; 12%) and was not provided for all cases from local congenital anomaly registers (13,085; 57%).

**Table 4:** The number of cases and adjusted overall ORs (95% CIs) for various congenital anomalies by TTHM category.

	Cleft lip palate		Abdominal wall defects		Major cardiac defects		NTDs		Urinary tract defects		Respiratory defects	
Category	No.	OR (95%CI)	No.	OR (95%CI)	No.	OR (95%CI)	No.	OR (95%CI)	No.	OR (95%CI)	No.	OR (95%CI)
TTHM												
Low	1,482	1.00	970	1.00	2,948	1.00	1,466	1.00	2,019	1.00	664	1.00
Medium	1,505	0.97 (0.88–1.05)	973	0.97 (0.87–1.08)	3,709	0.99 (0.82–1.20)	1,437	0.98 (0.85–1.13)	2,320	1.06 (0.91–1.23)	588	0.98 (0.86–1.11)
High	530	0.94 (0.83–1.06)	288	0.81 (0.68–0.95)	1,166	0.96 (0.78–1.17)	421	0.91 (0.73–1.13)	724	0.94 (0.78–1.14)	121	1.00 (0.80–1.26)
TBROM												
Low	1,242	1.00	769	1.00	2,543	1.00	1,226	1.00	1,718	1.00	535	1.00
Medium	1,570	0.98 (0.89–1.06)	1,035	1.03 (0.92–1.14)	3,957	1.12 (1.01–1.23)	1,446	0.98 (0.85–1.14)	2,412	1.09 (0.98–1.20)	574	1.13 (0.87–1.46)
High	705	0.96 (0.86–1.07)	427	1.02 (0.89–1.17)	1,323	1.13 (0.93–1.37)	652	0.90 (0.79–1.03)	933	1.05 (0.89–1.25)	264	1.09 (0.80–1.49)
Bromoform												
Low	2,206	1.00	1,401	1.00	5,263	1.00	2,074	1.00	3,361	1.00	896	1.00
Medium	697	1.06 (0.96–1.18)	438	1.01 (0.83–1.22)	1,326	1.07 (0.90–1.27)	663	1.01 (0.84–1.22)	921	1.01 (0.92–1.11)	291	1.07 (0.91–1.27)
High	614	1.01 (0.88–1.16)	392	1.15 (0.82–1.62)	1,234	1.04 (0.87–1.24)	587	0.96 (0.83–1.11)	781	0.96 (0.78–1.18)	186	0.98 (0.78–1.23)

TTHM category (low, < 30 μg/L; medium, 30–60 μg/L; high, > 60 μg/L), TBROM category (low, < 10 μg/L; medium, 10–20 μg/L; high, > 20 μg/L), and bromoform category (low, < 2 μg/L; medium, 2–4 μg/L; high, > 4 μg/L). Overall summary estimates were obtained from meta-analysis combining the registry-specific exposure ORs adjusted for Carstairs deprivation quintile, year, water company, and mother’s age. The meta-analyses incorporated random effects where necessary to allow for heterogeneity between registries.
